# A Novel Outbred Mouse Model of 2009 Pandemic Influenza and Bacterial Co-Infection Severity

**DOI:** 10.1371/journal.pone.0082865

**Published:** 2013-12-06

**Authors:** Kevin J. McHugh, Sivanarayana Mandalapu, Jay K. Kolls, Ted M. Ross, John F. Alcorn

**Affiliations:** 1 Department of Pediatrics, Children’s Hospital of Pittsburgh of UPMC, Pittsburgh, Pennsylvania, United States of America; 2 Richard K. Mellon Foundation Institute, Children’s Hospital of Pittsburgh of UPMC, Pittsburgh, Pennsylvania, United States of America; 3 Department of Microbiology & Molecular Genetics, University of Pittsburgh Center for Vaccine Research, Pittsburgh, Pennsylvania, United States of America; University of Tennessee Health Science Center, United States of America

## Abstract

Influenza viruses pose a significant health risk and annually impose a great cost to patients and the health care system. The molecular determinants of influenza severity, often exacerbated by secondary bacterial infection, are largely unclear. We generated a novel outbred mouse model of influenza virus, *Staphylococcus aureus*, and co-infection utilizing influenza A/CA/07/2009 virus and *S. aureus* (USA300). Outbred mice displayed a wide range of pathologic phenotypes following influenza virus or co-infection ranging broadly in severity. Influenza viral burden positively correlated with weight loss although lung histopathology did not. Inflammatory cytokines including IL-6, TNF-α, G-CSF, and CXCL10 positively correlated with both weight loss and viral burden. In S. aureus infection, IL-1β, G-CSF, TNF-α, and IL-6 positively correlated with weight loss and bacterial burden. In co-infection, IL-1β production correlated with decreased weight loss suggesting a protective role. The data demonstrate an approach to identify biomarkers of severe disease and to understand pathogenic mechanisms in pneumonia.

## Introduction

Influenza represents a highly contagious family of respiratory viruses that infect between 5-20% of the US population and account for as many as 30,000 deaths annually. However, influenza causes a broad range of disease outcomes ranging from very mild infection to death. The influenza A virus H1N1 has been the cause of two global pandemics over the last century. The 1918 H1N1 pandemic infected nearly one-third of the world population (about 500 million cases) with mortality rates greater than 2.5%, much higher than usual seasonal outbreaks (0.1%) [1]. Mortality during the 1918 influenza pandemic occurred primarily in two waves: first, an acute response to virus, resulting in excessive inflammatory mediator production and diffuse alveolar damage resulting in acute respiratory distress; second, for 20% of those infected, development of secondary bacterial pneumonia [2]. Recent work characterizing the 1918 influenza outbreak has identified secondary bacterial infection as a key contributor to mortality [3]. The primary pathogen involved in secondary pneumonia was *Streptococcus pneumoniae*. Similar co-infection data were observed during the recent 2009 novel H1N1 pandemic. Unlike seasonal influenza virus strains, novel H1N1 infections resulted in increased morbidity and mortality in young adults in addition to the very young and very old. In 2009 alone, approximately 61 million were infected in the US with 274,000 hospitalizations and 12,470 deaths according to the Center for Disease Control CDC (http://www.cdc.gov/h1n1flu/estimates_2009_h1n1.htm) [4]. In a CDC study of mortalities in young adults (median age 31), 29% were positive for secondary bacterial infection; among those, 45% were *S. pneumoniae* positive and 33% were *Staphylococcus aureus* positive [5]. 

Exacerbation of influenza A virus pneumonia by secondary *S. pneumoniae* or *S. aureus* infection in mice has been demonstrated [6,7]. Influenza virus infection has been shown to enhance susceptibility to secondary bacterial infections by multiple pathways including altering bacterial adhesions, TLR expression and pathogen-associated molecular pattern (PAMP) receptors on epithelial cells [8-11]. Induction of type I (IFNα and IFNβ) or type II (IFNγ) interferons by influenza A virus was shown to inhibit clearance of pneumococcal and *S. aureus* pneumonia in mice [12-14]. Recently, we and others have shown that preceding influenza virus infection suppresses subsequent T_H_17 pathway activation by secondary bacterial infection [12,15]. These data indicate a mechanism by which influenza A virus infection enhances the lung’s susceptibility to secondary bacterial pneumonia. Furthermore, many investigators have demonstrated a synergism between influenza A virus and *S. aureus* in lung injury [16-18]. Bacterial sepsis represents a significant cause of death in the United States (200,000 deaths annually) and the primary cause and location of sepsis is gram-positive bacteria in the lung [19,20]. Several studies have temporally linked the onset of influenza A virus infection and the incidence of *S. aureus* pneumonia [21]. Furthermore, the presence of preceding influenza-like symptoms was shown to correlate with increased mortality in methicillin-resistant *S. aureus* (MRSA) infected patients [22]. A recent study of children with complicated pneumonia showed that *S. aureus* was the most prevalent bacterial infection associated with influenza A virus and that this co-infection resulted in increased intensive care admission, ventilator usage, cost of stay, and mortality [23]. These data suggest that a primary severe consequence of influenza A virus infection is secondary *S. aureus* pneumonia.

Influenza virus infection has been modeled in mice for many years in order to gain insight into disease pathogenesis and the host response. The majority of these studies have been performed in inbred mouse lines that exhibit strain specific characteristics in response to influenza virus. While these studies have proven informative in many critical areas, the intrastrain uniformity of influenza virus infection has limited the determination of the molecular signature of severe disease. Outbred mouse lines have been utilized for influenza virus vaccine studies for the last 25 years. However, very little is known about the characteristics of severe versus mild disease in this context. In addition, there are many known problems regarding classic outbred mouse lines in terms of defining and tracking allelic diversity [24]. The goal of this study was to utilize a novel outbred mouse line (Jackson Laboratories Diversity Outbred) to examine both mild and severe influenza virus infection. With this approach, the critical hallmarks of severe influenza virus, *S. aureus*, and co-infection were examined. 

## Materials and Methods

### Animals

Age-matched male Diversity Outbred mice (8 to 10 weeks) were purchased from Jackson Laboratories (Bar Harbor, ME). In the initial influenza virus study, mice ranged in weight from 17.4 to 28.0 g. In the co-infection studies mice ranged in weight from 21.5 to 37.6 g. All animal studies were approved by the Institutional Animal Care and Use Committee of the University of Pittsburgh protocol number 1108659. All invasive procedures were performed under isoflurane or pentobarbital anesthesia to ameliorate animal distress.

### Influenza virus and bacterial infection

Mice were infected with 1x10^6^ pfu of influenza A/California/07/2009 virus by intranasal inoculation. Influenza virus was grown in chicken eggs and viral hemagglutinin and neuraminidase were sequenced to ensure no mutations. The influenza virus dose was chosen based on preliminary studies in C57BL/6 mice that resulted in 15-20% weight loss mimicking severe influenza virus infection. Mice were the harvested either five or eight days after influenza virus infection. In bacterial infections studies, mice received 1x10^8^ cfu of MRSA (USA300) by intranasal inoculation. Tissues were harvested two days after bacterial infection. For co-infection studies mice received influenza virus then were challenged with *S. aureus* six days after viral infection, mice were then harvested an additional two days later. All mice were tracked for morbidity by measuring weight loss daily post-infection. For influenza virus studies, “high” weight loss was defined as losing more than 15% initial weight while “low” weight loss refers to less than 5% weight loss. For *S. aureus* infected mice, “low” refers to less than 3% weight loss, high weight loss refers to greater than 5% loss from starting weight.

### Assessment of inflammation

On harvest day, the lungs were bronchoalveolar lavaged (BAL) with sterile PBS. BAL inflammatory cells were examined by total cell counts. BAL protein concentration was analyzed by bicinchoninic acid protein assay (Thermo Fisher Scientific, Rockford, IL). Serum was collected by transection of the inferior vena cava and renal artery into heparin treated tubes. The left lung was isolated and fixed with 10% neutral buffered formalin for histology. The upper right lung lobe was homogenized in PBS for bacterial colony counts, viral burden by qRT-PCR, and cytokine analysis by Lincoplex (Millipore, Billerica, MA). The middle and lower right lung lobes were snap frozen in liquid N_2_ for isolation of total RNA. 

### Gene expression analysis

Total RNA was isolated from lung tissue using the Absolutely RNA Miniprep Kit (Agilent, Santa Clara, CA). For qualitative real-time PCR analysis (qRT-PCR), cDNA was generated using an iScript cDNA Synthesis Kit (Biorad, Hercules, CA). qRT-PCR was performed using Assay On Demand primer and probe sets for target genes of interest (Life Technologies, Grand Island, NY) with the Applied Biosystems 7900 machine (Life Technologies). Gene expression was quantified using Hypoxanthine-guanine phosphoribosyltransferase (HPRT) as a lung housekeepeing gene. Data were converted to relative expression levels using the ΔΔCT (cycles to threshold) method. Influenza virus matrix gene expression was detected by using the following primers/probe - Forward primer: 5' GGACTGCAGCGTAGACGCTTT 3' Reverse primer: 5' CATCCTGTTGTATATGAGKCCCAT 3' Probe: 5' 6FAM-CTMAGYTATTCWRCTGGTGCACTTGCC-BHQ 3'. The term “high viral burden” refers to mice with a 10-fold elevated matrix gene expression versus a “low burden” animal in the study group and represents relative changes in burden. 

### Histologic analyses

Formalin-fixed lung tissue was embedded in paraffin and cut into 5 μm sections. Sections were then stained with hematoxylin and eosin for analysis. Histologic scoring of inflammation was performed by a trained pathologist. Slides were blinded and scored for parenchymal, perivascular, and peribronchial inflammation using a 1 to 4 scale with 4 being the most severe.

### Statistics

Correlation analysis was performed using Pearson’s product-moment correlation coefficient (R) to determine the degree of significance using the Microsoft Excel software package (Redmond, WA). Statistical significance was set at p-value < 0.05.

## Results

### Generation of a novel mouse model of pandemic influenza virus severity

In order to best examine the molecular phenotype of influenza virus infection severity in mice, we chose to develop a novel model for influenza virus infection in a newly available outbred mouse line. To do this, we utilized Jackson Laboratories Diversity Outbred (JDO) mice. Jackson Laboratories has recently introduced this novel line as an improvement on existing outbred colonies. JDO mice were produced by a novel outbreeding strategy designed to preserve founder genomes and avoids allelic loss. The JDO founders were derived from mice from the Collaborative Cross (CC) [25]. The CC is a large group of inbred mouse strains derived from an eight-way cross using a set of mice that included three wild-derived strains. Recent work using the CC mice has examined severity markers in the context of mouse influenza (A/PR/8/34) virus infection [26,27]. We have chosen the JDO model herein to maximize allelic variation throughout the genome. Another advantage is that Jackson Laboratories offers an array chip to map over 620,000 single nucleotide polymorphisms (SNP). This would allow us in the future to perform SNP analysis on mice with interesting phenotypes in response to influenza virus and/or co-infection. 

**Figure 1:**
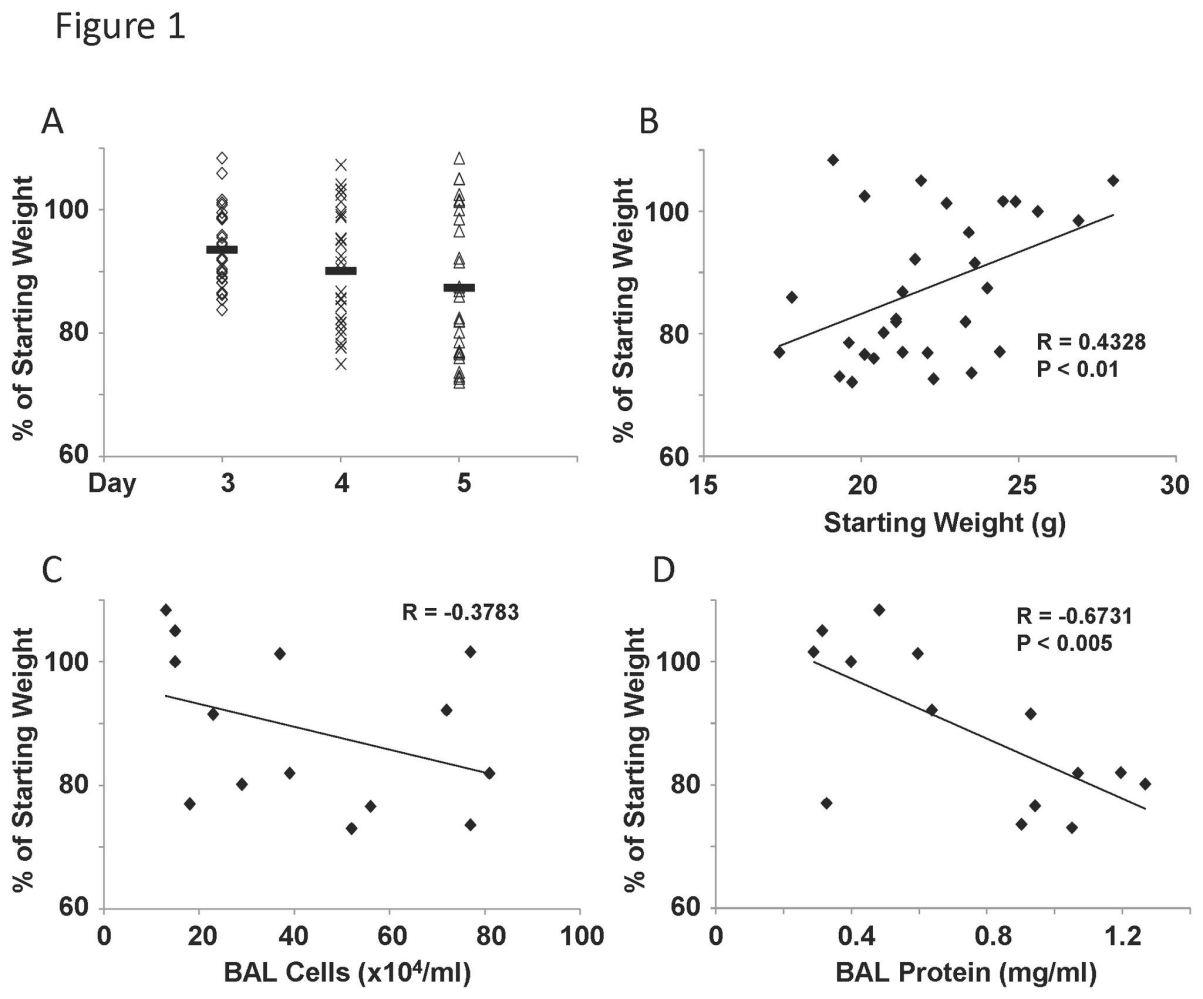
Influenza virus induces variable weight loss and inflammation in outbred mice. Diversity outbred mice were infected with 1x10^6^ pfu of influenza A/California/07/2009 virus and were harvested five days later. A, weight loss over time as percentage of pre-challenge starting weight (N=30). B, correlation between starting weight and weight loss percentage (N=30). C, bronchoalveolar lavage cell counts correlated with weight loss percentage (N=14). D, bronchoalveolar lavage protein concentration correlated with weight loss percentage (N=14). R, Pearson’s Rho; P indicates significance as listed. Solid bar indicates group mean.

We infected 30 male JDO mice with influenza virus (pandemic H1N1 CA/07/2009, 1x10^6^ pfu, intranasally) for five days. Weight loss was used as a primary endpoint for morbidity. Inbred mouse strains generally provide limited variation between animals. JDO mice displayed a much larger range of weight loss phenotypes, from 108.4 to 73.0% of starting body weight (Figure 1A). We observed clear populations of mice that had very little or no weight loss. By day five, 10 of 30 mice had greater than 95% of the original body weight. On the other hand, 11 of 30 mice were below 80% of their initial weight. These findings indicate clear groupings of mice into mild and severe phenotypes and confirm that the JDO mouse line had a variable response to influenza virus infection. Weight loss on day 3 significantly correlated with weight loss in the same animal by day five (R = 0.888, p < 0.005). Next, we examined if starting weight had an impact on eventual influenza outcome. We saw a significant positive correlation between starting weight and weight loss percentage (Figure 1B). The mice used in the study were age matched 8 week old mice. These data suggest that larger, heartier animals would be predicted to suffer reduced morbidity compared to smaller animals. From the original 30 mouse study, we harvested 14 mice on day five for further analysis. We compared bronchoalveolar lavage (BAL) cell counts and protein concentrations with weight loss percentage. Interestingly, BAL total inflammatory cell counts did not significantly correlate with weight loss (Figure 1C). However, BAL protein concentration significantly negatively correlated with starting weight percentage (Figure 1D). These data indicate that increased BAL protein concentration is predictive of increased weight loss and thus morbidity. 

### Weight loss and viral burden significantly correlate

**Figure 2:**
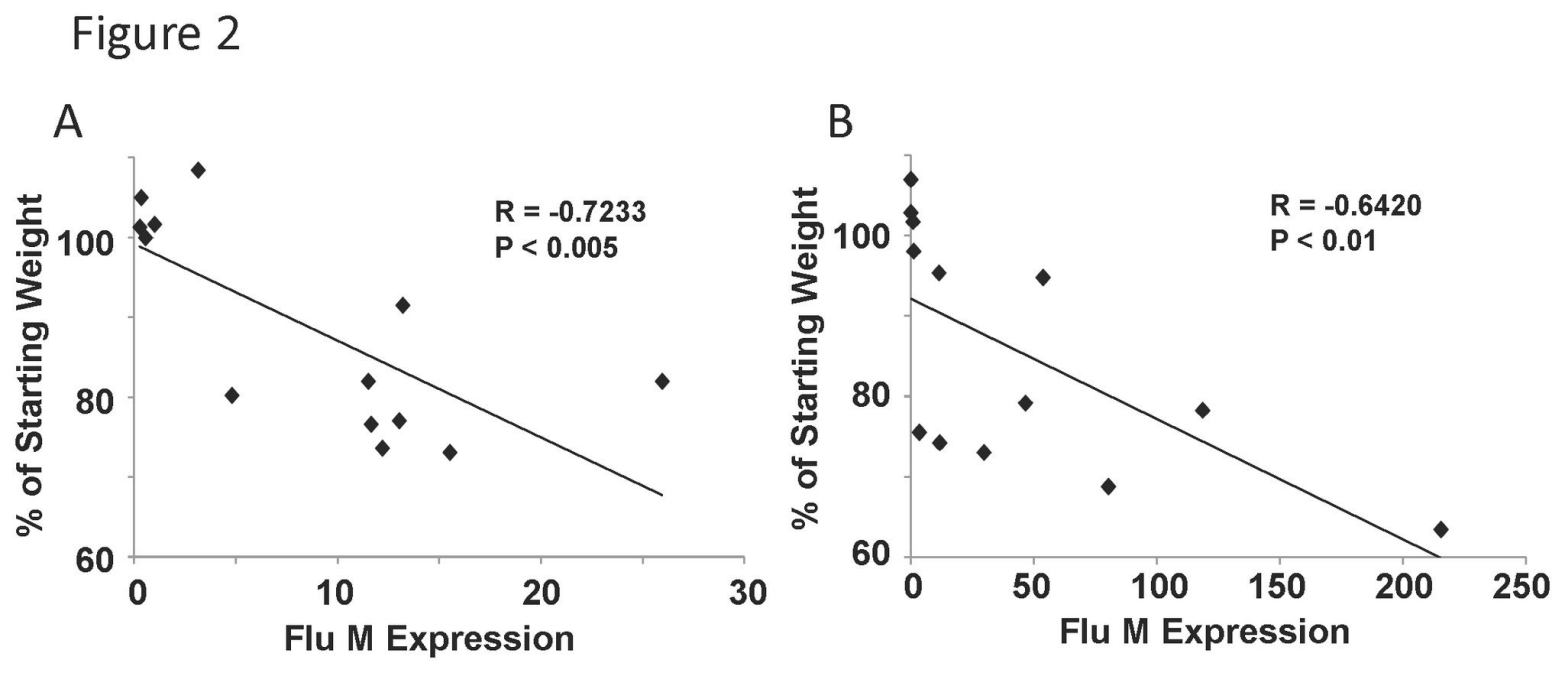
Viral burden correlates with weight loss in outbred mice. Diversity outbred mice were infected with 1x10^6^ pfu of influenza A/California/07/2009 virus and were harvested five or eight days later. A, day five influenza virus M protein gene expression (determined by qRT-PCR) correlated with weight loss percentage (N=13). B, day eight influenza virus M protein gene expression correlated with weight loss percentage (N=13). R, Pearson’s Rho; P indicates significance as listed.

A possible explanation for the differences seen in weight loss is variable viral burden in mice. RNA was isolated from influenza virus infected lungs for qRT-PCR analysis. Influenza virus burden was determined by qRT-PCR for viral matrix protein specific for H1N1 CA/07/2009. Viral M protein RNA was detectable in all mice in the study on both day 5 and 8 post infection. We found a significant correlation between weight loss and viral burden (Figure 2), both five and eight days after infection. Despite this correlation, there were examples of mice with similar viral burden, but disparate weight loss. Thus, viral burden alone does not explain the range of morbidity phenotypes observed. 

### Lung histopathology demonstrates a broad range of disease phenotypes

**Figure 3:**
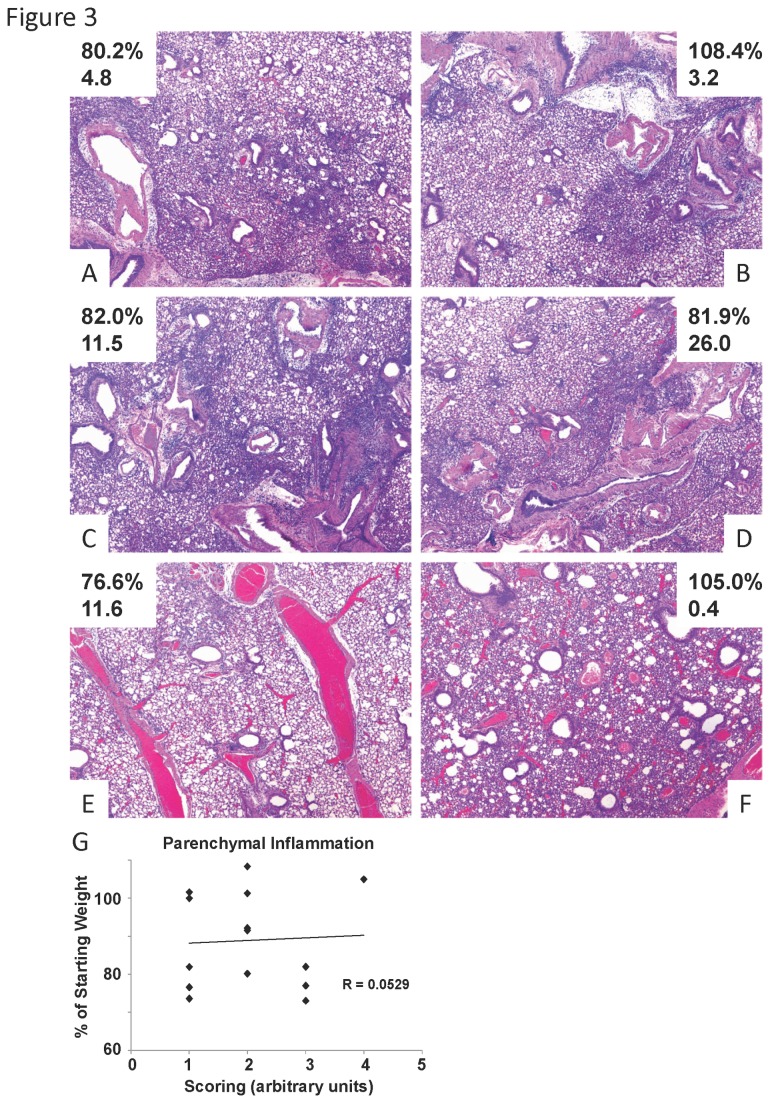
Variable lung pathology in influenza virus infected outbred mice. Diversity outbred mice were infected with 1x10^6^ pfu of influenza A/California/07/2009 virus and were harvested five days later. A, B, individual mice with similar influenza viral burden (relative M protein gene expression, lower number) but disparate weight loss (weight loss percentage, upper number). C, D, individual mice with similar weight loss but different viral burdens. E, individual mouse with extreme weight loss and high viral burden, but with minimal lung inflammation pathology. F, individual mouse with no weight loss and low viral burden, but with significant lung injury. G, histologic scoring of lung parenchymal inflammation.

In order to determine if weight loss and/or viral burden directly related to lung pathogenesis, lungs were fixed and analyzed by light microscopy. Interestingly, the degree of lung injury observed was variable and did not directly reflect weight loss or viral burden at day five post infection. Mice with similar viral burden, but different weight loss displayed similar amounts of parenchymal inflammation (Figure 3A,B). Further, mice with similar weight loss, but different viral burden also showed similar amounts of parenchymal inflammation (Figure 3C,D). In addition to these findings, individual mice often had surprising histologic phenotypes. For example, a mouse with severe weight loss and high viral burden had less lung inflammation (Figure 3E). Conversely, a mouse with no weight loss and very low viral burden had significant parenchymal consolidation (Figure 3F). Further, histologic lung inflammation (parenchymal, R = 0.0529; peribronchial, R = -0.3413; perivascular, R = -0.1428) did not significantly correlate with weight loss (Figure 3G). In addition, lung inflammation did not correlate with viral burden (parenchymal, R = -0.1304; peribronchial, R = 0.2827; perivascular, R = 0.2161). These data demonstrate that diverse lung pathologic phenotypes following influenza virus do not directly correlate to weight loss (morbidity) or viral burden. In addition, this range of phenotypes displayed in this novel model may better reflect the diversity of human responses to influenza virus. 

### Cytokine production correlates with weight loss and viral burden

**Figure 4:**
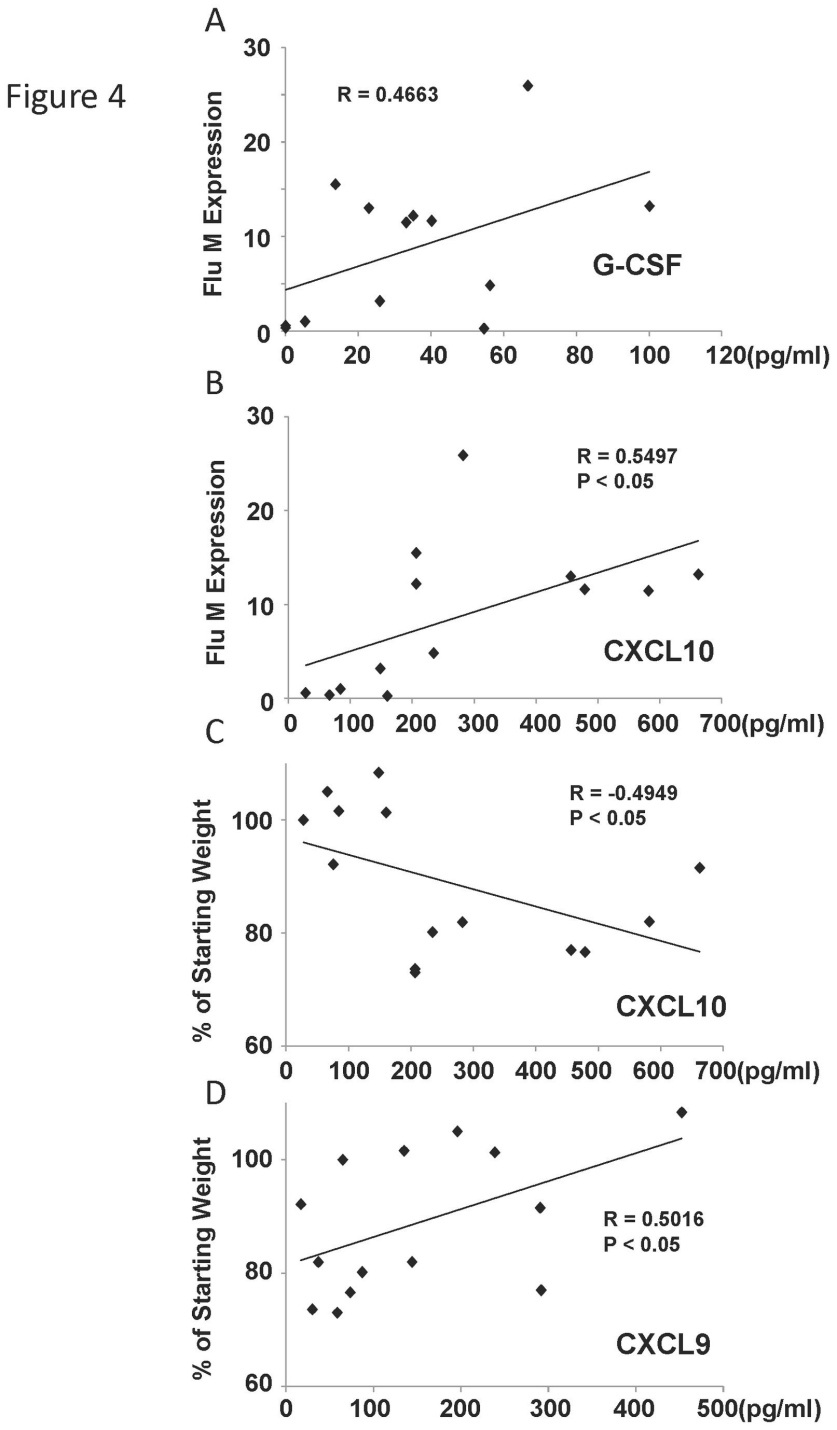
Inflammatory cytokine levels correlated with influenza viral burden or weight loss. Diversity outbred mice were infected with 1x10^6^ pfu of influenza A/California/07/2009 virus and were harvested five days later. A, cytokine levels in lung homogenate (by Lincoplex) correlated with influenza virus M protein gene expression (by qRT-PCR) (N=13). B, cytokine levels in serum correlated with influenza virus M protein gene expression (N=14). C, D, cytokine levels in serum correlated with weight loss percentage (N=14). R, Pearson’s Rho; P indicates significance as listed.

Lung and serum cytokine levels may serve as a biomarker for influenza severity. To examine this, we first prepared lung homogenate from the day five influenza virus infected mice and analyzed inflammatory cytokines by Lincoplex. We measured 19 cytokines and did not find a significant correlation between any cytokine and weight loss percentage. However, we did detect a trend towards a positive correlation between G-CSF and viral burden (Figure 4A). Next, we examined serum cytokine levels and determined that CXCL10 (IP-10) significantly correlated with viral burden (positive) and starting weight percentage (negative) (Figure 4B,C). Interestingly, another CXCR3 ligand, CXCL9, positively correlated with starting weight percentage (Figure 4D). These data show that a limited number of serum cytokines may be linked to influenza morbidity and burden. 

### Inflammatory gene expression in the lung correlates with weight loss and viral burden

**Figure 5:**
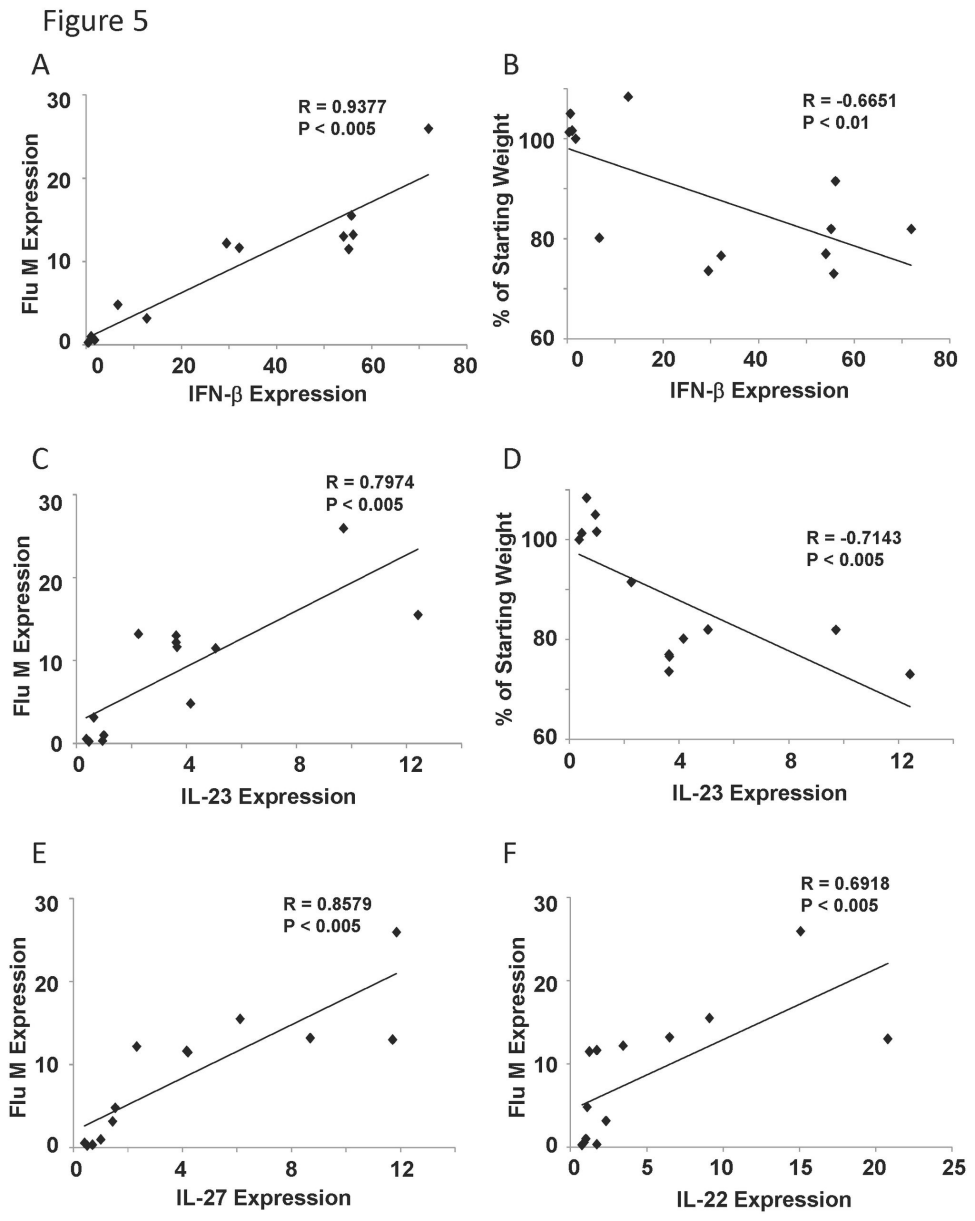
Cytokine gene expression correlated with influenza viral burden or weight loss. Diversity outbred mice were infected with 1x10^6^ pfu of influenza A/California/07/2009 virus and were harvested five days later. A, C, E, F, influenza virus M protein gene expression correlated with cytokine expression (by qRT-PCR) (N=13). B, D, weight loss percentage correlated with cytokine expression (N=13). R, Pearson’s Rho; P indicates significance as listed.

A primary host response to viral infection is production of type I interferon (IFN-α/β). To examine if type I interferon is linked with viral burden or weight loss, lung RNA was analyzed by qRT-PCR. Indeed, IFN-β and IL-27 (another STAT1 agonist) expression were strongly positively correlated with viral burden in the lung (Figure 5A,E). Further, IFN-β and IL-27 production negatively correlated with starting weight percentage (Figure 5B, data not shown). Our laboratory has recently shown that IL-17 plays a critical role in lung injury induced by influenza virus [28]. T_H_17 pathway cytokines (IL-17, IL-22, and IL-23) were examined by qRT-PCR. IL-17 gene expression was poorly detected in lung RNA at day five post-infection (data not shown). IL-23 expression significantly correlated with viral burden and starting weight percentage (Figure 5C,D). In addition, IL-22 expression positively correlated with viral burden, but not weight loss (Figure 5F). These data demonstrate that inflammatory cytokine gene expression can be correlated with both viral burden and weight loss during influenza virus infection.

### Inflammatory cytokines in the lung correlate with morbidity later during infection

**Figure 6:**
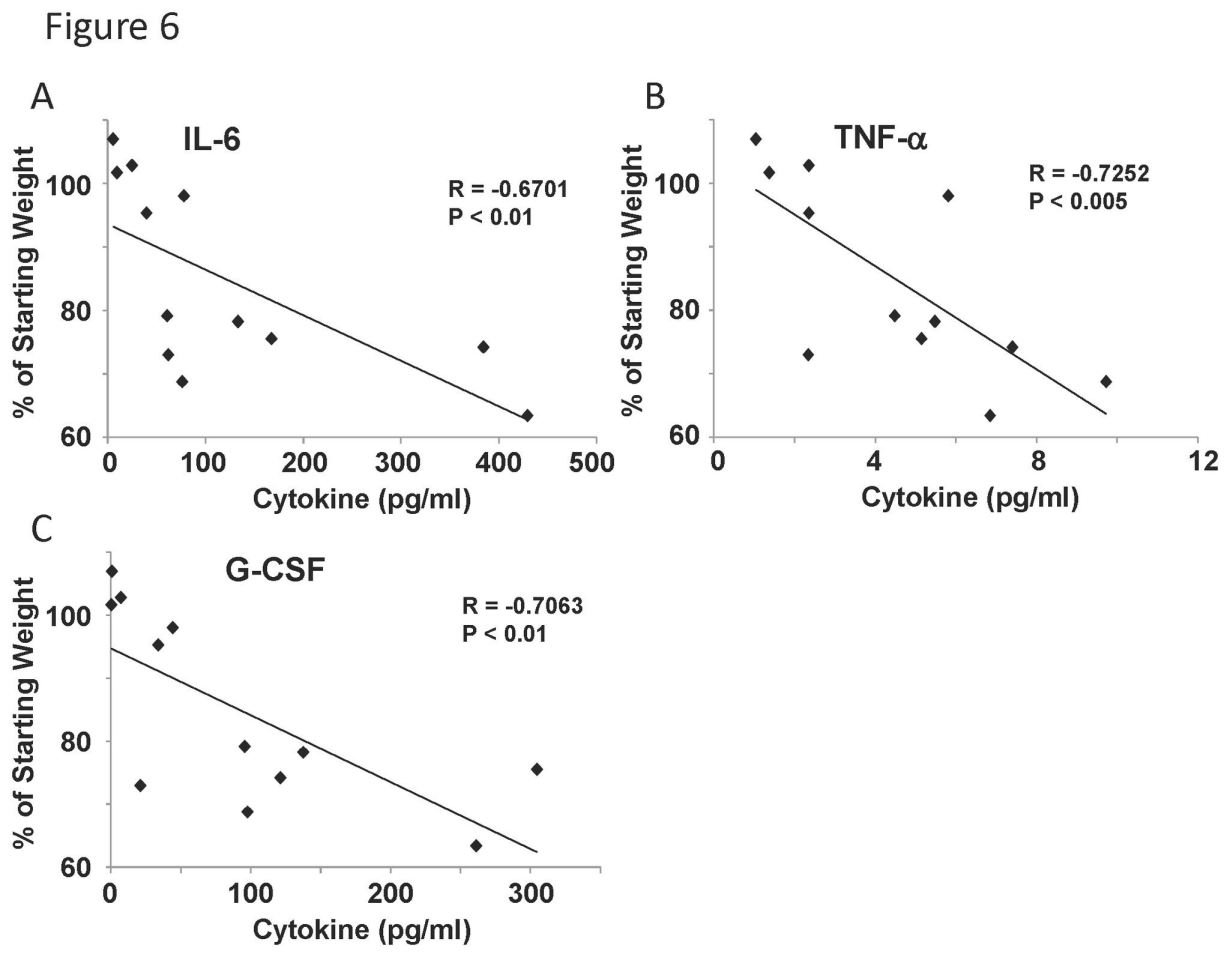
Inflammatory cytokine levels correlated with influenza virus induced weight loss at day eight. Diversity outbred mice were infected with 1x10^6^ pfu of influenza A/California/07/2009 virus and were harvested eight days later. A-C, weight loss percentage correlated with lung homogenate cytokine concentration (by Lincoplex) (N=12). R, Pearson’s Rho; P indicates significance as listed.

Since no correlation between lung cytokines and weight loss or viral burden were found at day five post infection, we followed another set of mice for eight days post infection and examined inflammatory cytokines in the lung homogenate. Eight days after infection there was no correlation between BAL inflammation and weight loss, similar to findings at day five. However, at day eight, a broad range of cytokines and chemokines correlated with weight loss. A highly significant negative correlation was observed between starting weight percentage and chemokines, such as MIP-1α, CXCL9, CXCL10, MCP-1, and KC (data not shown). These chemokines were most elevated in mice that lost the most weight. In addition, we detected significant negative correlation with TNF-α, G-CSF, and IL-6 (Figure 6). These data identify cytokine biomarkers of worsened disease morbidity during influenza A virus infection. Influenza viral burden significantly positively correlated with TNF-α, G-CSF, IL-6, MIP-1α, CXCL10, MCP-1, and KC (data not shown). This finding demonstrates a relationship between persistent lung inflammation and elevated viral burden during infection.

### Generation of a novel mouse model of *S. aureus* pneumonia and influenza virus co-infection

**Figure 7:**
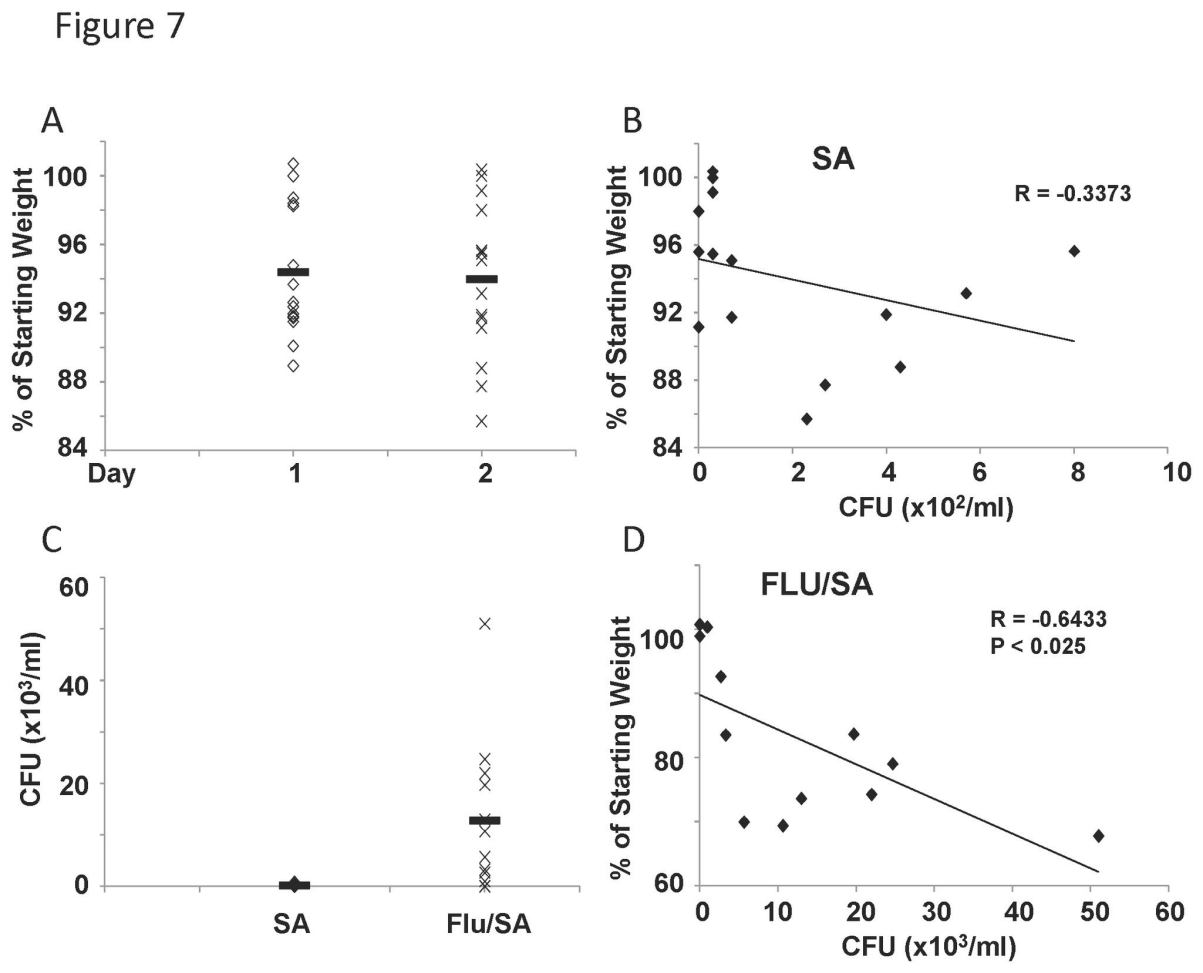
Influenza virus, *S. aureus* co-infection results in variable bacterial burden in outbred mice. Diversity outbred mice were infected with 1x10^6^ pfu of influenza A/California/07/2009 virus or vehicle control followed by infection with *S. aureus* USA300 on day 6 and then harvested 2 days later. A, weight loss induced by *S. aureus* infection alone in outbred mice (N=15). B, weight loss induced by *S. aureus* alone correlated with bacterial burden in lung homogenate (N=15). C, bacterial burden in the lungs of *S. aureus* only and co-infected mice (N=15, 12). D, weight loss induced by *S. aureus*, influenza virus co-infection correlated with bacterial burden in lung homogenate (N=12). R, Pearson’s Rho; P indicates significance as listed. Solid bar indicates group mean.

Since the majority of mortality observed in previously healthy individuals infected with influenza virus involves bacterial co-infection, we first established a novel model of *S. aureus* pneumonia in JDO mice. Mice were challenged with *S. aureus* (USA300, 10^8^ cfu, intranasally) for forty-eight hours. *S. aureus* induced variable weight loss in outbred mice (Figure 7A). Weight loss and bacterial burden in the lung trended to, but did not significantly correlate (Figure 7B). These data indicate the JDO model likely provides a broad range of phenotypes in response to *S. aureus* challenge, similar to influenza virus infection. 

Our laboratory has recently published an influenza virus, *S. aureus* co-infection model [12]. Mice were infected with influenza (pandemic H1N1 CA/07/2009, 1x10^6^ pfu) virus for 6 days, then they were challenged with *S. aureus* (USA300, 10^8^ cfu, intranasally) for 2 additional days. On day eight, tissues were harvested and bacterial colonization of the lung was determined by plating lung homogenate and colony counts. JDO mice challenged with only *S. aureus* had relatively low lung colony counts (ranging from 0 to 5.7 x 10^2^ CFU/ml), while co-infected mice had significant exacerbation of bacterial burden (Figure 7C). In co-infected mice, weight loss and bacterial burden significantly correlated (Figure 7D). These data demonstrate a variable phenotype in response to co-infection, with some mice having high bacterial load following influenza virus infection, while others with substantially lower burden. 

### Lung histopathology demonstrates a broad range of *S. aureus* infection phenotypes

**Figure 8:**
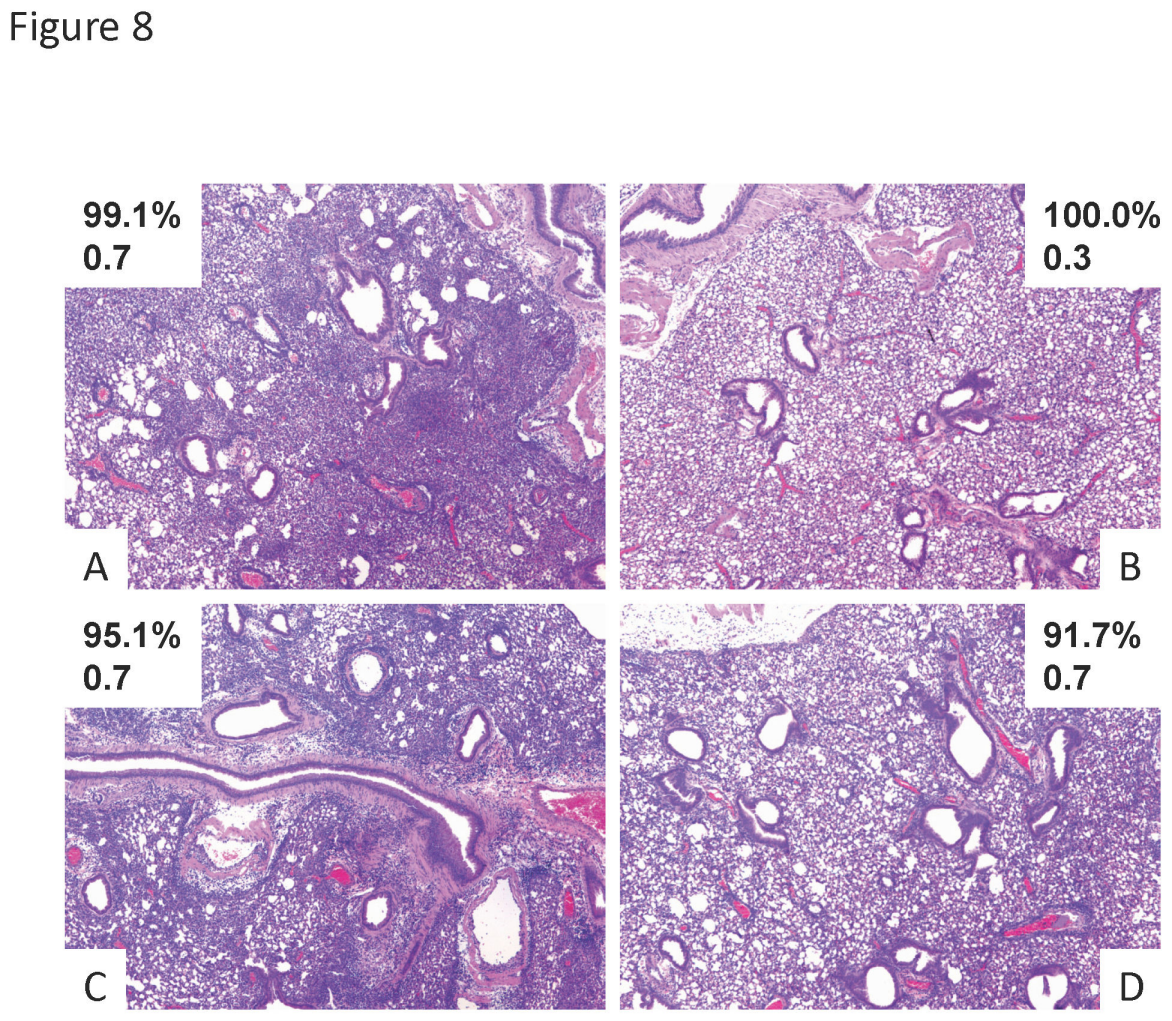
*S. aureus* infection results in variable lung pathology in outbred mice. Diversity outbred mice were infected *S. aureus* USA300 and then harvested 2 days later. A-D, lung pathology from individual mice with weight loss percentage (upper number) and *S. aureus* burden (lower number, x10^3^ cfu/ml).

Since JDO mice infected with *S. aureus* showed differential weight loss and bacterial burden, we examined lung pathology in these mice. Similar degrees of low weight loss were characterized by very different amount of lung inflammation (Figure 8A,B). A range of diffuse inflammation to little or no inflammation was observed. Further, severe inflammation was seen in a mouse with low bacterial burden (Figure 8C) and low levels of inflammation were present in a mouse with relatively high weight loss (Figure 8D). These examples illustrate a broad spectrum of lung pathology phenotypes in the JDO mouse model. 

### Lung cytokine production correlates with weight loss and bacterial burden

**Figure 9:**
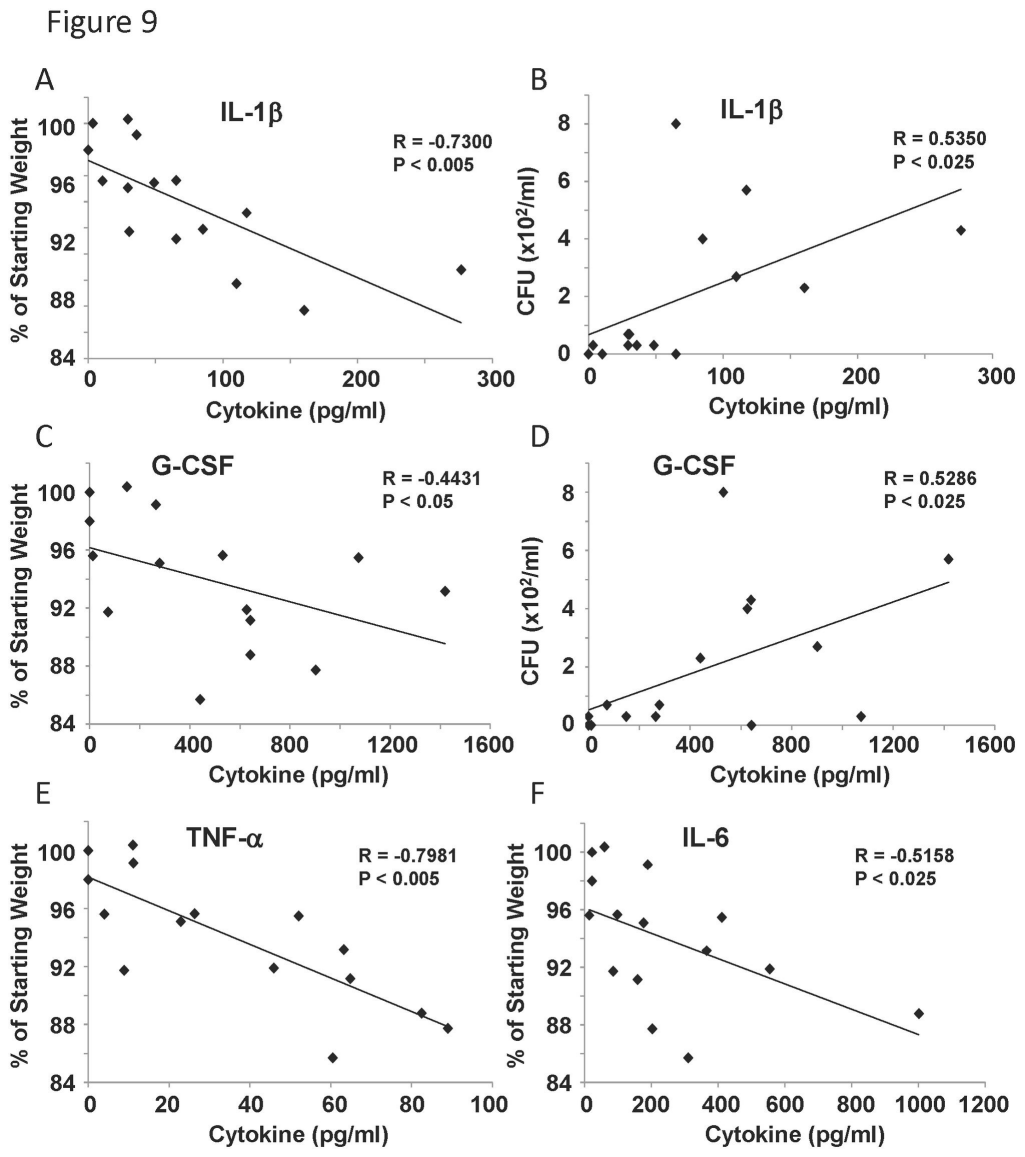
Inflammatory cytokine levels correlated with weight loss or bacterial burden in *S. aureus* infected mice. Diversity outbred mice were infected *S. aureus* USA300 and then harvested 2 days later. A, C, E, F, weight loss percentage correlated with cytokine expression in lung homogenate (by Lincoplex) (N=15). B, D, bacterial burden correlated with cytokine expression in lung homogenate (N=15). R, Pearson’s Rho; P indicates significance as listed.

Analysis of cytokines in lung homogenate from *S. aureus* infected mice revealed numerous correlations between weight loss and cytokine concentration. Once again, we detected that elevation of chemokines MIP-2, CXCL9, CXCL10, MIP-1α and KC (data not shown) significantly correlated with increased weight loss. In addition, IL-6, IL-1β, TNF-α, and G-CSF significantly correlated with decreased starting weight (Figure 9A,C,E,F). These data confirm that increased inflammatory mediators correlate with increased weight loss. Further, both IL-1β and G-CSF (as well as CXCL9 and MIP-1α, data not shown) positively correlated with bacterial burden (Figure 9B,D). These data identified several inflammatory mediators that track with both morbidity and bacterial burden during *S. aureus* lung infection. 

### Lung cytokine production correlates with weight loss and viral burden in an influenza virus, *S. aureus* co-infection model

**Figure 10:**
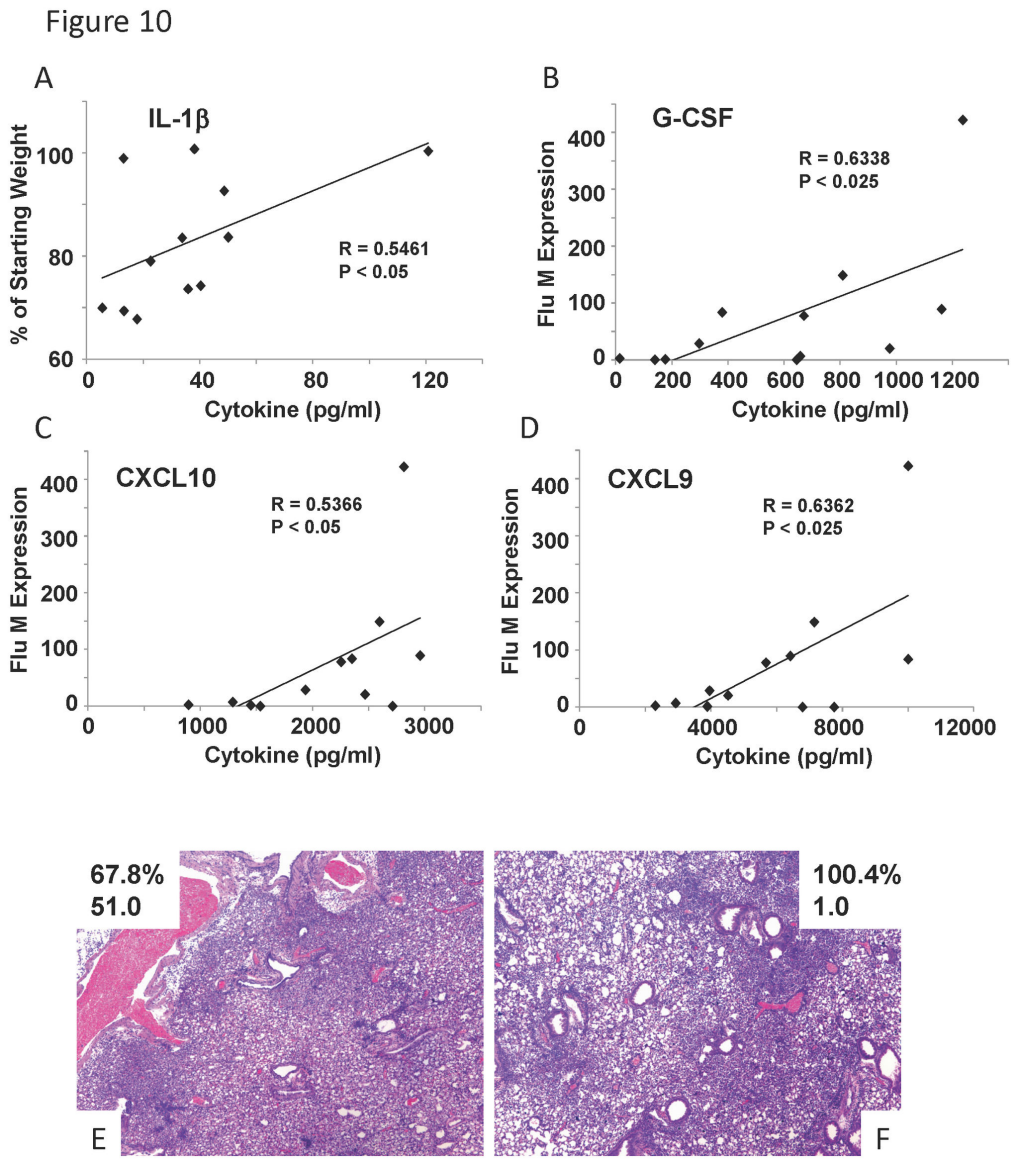
Inflammatory cytokine levels correlated with weight loss or viral burden in influenza virus, *S. aureus* co-infected mice. Diversity outbred mice were infected with 1x10^6^ pfu of influenza A/California/07/2009 virus followed by infection with *S. aureus* USA300 on day 6 and then harvested 2 days later. A, weight loss percentage correlated with cytokine expression in lung homogenate (by Lincoplex) (N=12). B-D, influenza virus M protein gene expression correlated with cytokine expression in lung homogenate (N=12). E, F variable pathology in co-infected individual mice. R, Pearson’s Rho; P indicates significance as listed.

In our co-infection model described above, weight loss did not significantly correlate with BAL inflammatory cells, similar to influenza virus or *S. aureus* infection alone (data not shown). Further, in the context of co-infection, viral burden and weight loss did not significantly correlate (data not shown). To determine inflammatory markers of severity, we examined cytokine levels in influenza A virus, *S. aureus* co-infected mice. Interestingly, we detected only one cytokine, IL-1β that was significantly positively correlated with starting weight percentage (Figure 10A). These data demonstrate that elevated IL-1β levels correlate with less morbidity during co-infection, suggesting a protective role for IL-1β in this model. Indeed, our previous work has demonstrated that preceding influenza virus infection attenuates the IL-1β responses to *S. aureus* alone in inbred mice perhaps increasing susceptibility [12]. 

Lung cytokines during co-infection did not significantly correlate with bacterial burden in the lung unlike the data with *S. aureus* infection alone (data not shown). Similar to day eight influenza virus infected mice alone, co-infected mice displayed correlation between cytokine levels and viral burden in co-infected animals. Influenza viral burden significantly positively correlated with G-CSF, IL-10, MIP-1α, CXCL9, CXCL10, MCP-1, and KC production (Figure 10B-D, data not shown). Analysis of lung histopathology demonstrated a wide range of lung injury phenotypes. These included severe parenchymal inflammation in a mouse with high weight loss and viral burden (Figure 10E) and a mouse with no weight loss and low viral burden (Figure 10F). This illustrates the severity of lung phenotype in co-infection despite differences in morbidity which may reflect variable responses to these pathogens in humans. 

## Discussion

In this study, we demonstrate the utility of using a novel outbred mouse line to study the molecular phenotype of viral and bacterial pneumonia severity. By using a genetically diverse animal population, we can make correlative inferences relating to the hallmarks of severe disease pathogenesis. This approach is particularly useful to define determinants, or biomarkers, of severe disease that may relate to morbidity and mortality. It is also possible to gain mechanistic insight into immune host defense pathways by examining markers of improved clearance or decreased morbidity. Herein, we demonstrate that influenza virus causes a broad range of disease phenotypes in outbred mice in terms of weight loss (morbidity), inflammation, and tissue injury. Thus, this model reproduces many of the hallmarks of influenza illness in human populations. Interestingly, we found a significant correlation between animal weight and eventual weight loss. Larger animals lost less weight than smaller mice suggesting that stature and/or frailty may impact disease outcome. It is known in humans that the very young and elderly are often most susceptible to influenza virus induced mortality. We show that viral burden and morbidity correlate during the infection time course. Differences in viral burden between outbred mice exposed to matching inoculum may be due to many factors. Beyond the immune parameters measured herein, it is intriguing to speculate that microbiome differences may play a role in influenza pathogenesis. The role of the microbiome in directing T cell responses is rapidly emerging. At day five post-infection, CXCR3 chemokines in serum correlate with morbidity and viral burden. Interestingly, the degree of lung histopathologic injury is not directly linked to viral burden or morbidity. Further, STAT1/2 agonists and T_H_17 pathway gene expression correlate with both morbidity and viral burden. Later during infection, day eight, many inflammatory cytokines correlate with weight loss and viral burden suggesting that persistent cytokine production only occurs in severe disease. 

During the recent novel H1N1 pandemic several studies were conducted in an attempt to analyze serum cytokines in relationship to disease severity [29-31]. The primary consistent finding in these studies was a positive correlation between IL-6 levels and influenza severity. Our mouse study also identified persistent IL-6 production to be significantly correlated with increased morbidity. IL-6 has been shown to be important for influenza virus host defense in mouse models. IL-6 receptor -/- and IL-6 -/- mice displayed increased neutrophil death, worsened lung injury, and increased viral persistence compared to WT mice [32]. Additionally, IL-6 -/- mice had impaired CD4^+^ T cell memory responses to influenza virus, perhaps due to excessive activation of regulatory T cells [33]. These data suggest that blocking IL-6 during influenza virus infection is not likely to be an effective therapeutic approach. However, the degree of IL-6 production may be a useful biomarker of severe disease progression. In addition to IL-6, human serum studies identified TNF-α to be positively correlated with influenza severity. Our mouse model also showed elevated TNF-α significantly correlated with increased morbidity. These findings further support the usefulness of this model to study influenza severity. In addition to morbidity, both IL-6 and TNF-α levels positively correlated with influenza viral burden. It is likely that persistent inflammation driven by these pathways is a relevant biomarker for not only pathologic phenotype, but also viral persistence.

Our study also identified several correlations between morbidity or viral burden and T_H_17 pathway cytokines. Expression of IL-23, a key T_H_17/IL-17 promoting cytokine was positively correlated with influenza viral burden and weight loss. We found a similar expression pattern for IL-22, a cytokine produced by T_H_17, and other cells. Further, persistent G-CSF production in the lung, a pro-neutrophilic cytokine downstream of IL-17, was positively correlated with both morbidity and viral burden. IL-17 is known to promote lung pathology associated with influenza virus infection [28]. In fact, IL-17 receptor -/- mice were protected from influenza virus induced weight loss and inflammation, but did not display impaired viral clearance. These findings were confirmed in a 2009 H1N1 model [34]. Conversely, IL-22 has been shown to be protective during influenza virus infection [35-37]. In those studies, IL-22 protects the epithelium from influenza virus induced cell death and damage, promoting regeneration and lung repair. It is possible that T_H_17 pathway activation is elevated in severe influenza and that IL-17 may be detrimental while IL-22 is protective. 

Assessment of serum cytokines revealed only two chemokines that significantly correlated with influenza virus induced morbidity. Interestingly, we found that the CXCR3 ligands, CXCL9 and CXCL10 were related to weight loss and viral burden. CXCL10 was positively correlated with morbidity and influenza virus levels, while CXCL9 was negatively correlated with weight loss. Serum CXCL10 was shown to be positively correlated with 2009 H1N1 severity in humans [29]. Mouse studies have shown that attenuation of CXCR3 by either knockout or antibody treatment results in protection against influenza virus induced morbidity and mortality [38-40]. These data suggest that CXCR3 signaling is critical to lung pathogenesis during influenza virus infection. Our mouse model was able to identify this pathway as a biomarker of severe influenza. Somewhat surprisingly, we did not find a correlation between lung histopathology and weight loss or viral burden. It is possible that the host pathologic response to influenza virus is highly variable due to the combined impact of many pathways in addition to inflammatory mediators. These include epithelial injury genes, endoplasmic reticulum and mitochondrial stress pathways, and apoptotic cascades.

In this study, we also examined production of the STAT1/2 pathway activators; type I interferon and IL-27. We found that these cytokines positively correlated with viral burden, an expected finding as they are primary viral host defense genes. They also significantly correlated with weight loss. The interferon pathway and its downstream gene targets are well described in influenza virus infection [41]. Specifically, type I interferon promotes cytotoxic T cell lytic activity and B cell responses during influenza virus infection [42,43]. In fact, influenza virus NS1 protein targets the type I interferon pathway as a pathogenic mechanism [44]. Work in our laboratory and others have shown that elevated type I interferon may play a vital role in increasing the lung’s susceptibility to secondary bacterial pneumonia [12,13]. During severe influenza virus infection, elevated type I interferon leads to exacerbated bacterial infection through suppression of subsequent Type 17 immunity [12]. While interferon signaling may be beneficial in response to virus, it may be detrimental in terms of secondary pneumonia, which often results in poor outcomes. 

In addition to providing a novel viral infection model, outbred mice were utilized to examine *S. aureus* lung infection. Similar to influenza virus infection, outbred mice displayed a variable phenotype in terms of bacterial burden, weight loss, and lung injury. We also observed a disjoint between weight loss, bacterial burden, and lung histopathology. Production of the pro-inflammatory cytokines IL-6, TNF-α, and G-CSF all positively correlated with weight loss. G-CSF also positively correlated with bacterial burden in the lung. As discussed in the context of influenza virus infection above, these cytokines may serve as predictive biomarkers of severe bacterial pneumonia. We also identified IL-1β as having a positive correlation with weight loss and bacterial burden. Indeed, in a study of human community acquired pneumonia, IL-1β was a predictive marker for bacterial infection [45]. This is consistent with our findings that IL-1β tracks with bacterial infection and not influenza virus. In a cutaneous *S. aureus* infection model, IL-1β -/- mice had increased bacterial burden and decreased neutrophil recruitment [46]. Recent work by our group has shown that IL-1β is critical to host defense against S. aureus lung infection [47]. Our mouse model once again served as an accurate representation of the phenotype of human pneumonia.

Finally, we utilized the outbred mouse model to model influenza virus, *S. aureus* co-infection. We based our approach on our previously published inbred mouse work [12]. Interestingly, weight loss and viral burden did not correlate in co-infection, but bacterial burden and morbidity did correlate. In the current study, we showed that CXCR3 ligands and G-CSF positively correlated with viral burden, similar to influenza virus infection alone. However, cytokine production did not correlate with bacterial burden as it did in *S. aureus* infection alone. These data indicate that the complexity of co-infection may significantly alter the biomarkers associated with disease severity. We did show that the ability to generate an IL-1β response during co-infection negatively correlated with weight loss, suggesting that IL-1β is protective in this model. We have previously shown that preceding influenza virus infection suppresses *S. aureus* induced IL-1β levels [12]. This finding implies that mice that retain IL-1β in response to secondary *S. aureus* have lower morbidity. IL-1β levels were not detectable in serum from infected mice suggesting local production at the site of inflammation. This finding may complicate the use of this cytokine as a clinical biomarker although the potential to measure IL-1β in sputum is intriguing. Lung histopathology in co-infection remains highly variable between animals and neither bacterial burden nor weight loss are predictive of lung pathologic injury.

In this study, we present a novel outbred mouse model of 2009 H1N1 infection, *S. aureus* pneumonia, and co-infection. The utility of this model is many fold since we can learn about the molecular determinants of severe disease in a genetically diverse population. This information can be applied to designing novel biomarker approaches for human disease. In addition, we can unravel the mechanisms of inflammation and lung injury and further understand conserved and disjointed pathways of disease. For these reasons, the outbred mouse model can be utilized to address questions about influenza pathogenesis that are left unanswered by current approaches.
